# ‘Familiarity’ as a key factor influencing rural family carers’ experience of the nursing home placement of an older relative: a qualitative study

**DOI:** 10.1186/1472-6963-13-252

**Published:** 2013-07-03

**Authors:** Assumpta Ryan, Hugh McKenna

**Affiliations:** 1 School of Nursing and Institute of Nursing and Health Research, University of Ulster, Cromore Road, Coleraine, Co. Londonderry BT52 1SA, Northern Ireland; 2 Institute of Nursing and Health Research, University of Ulster, Cromore Road, Coleraine, Co. Londonderry BT52 1SA, Northern Ireland

**Keywords:** Family carers, Nursing homes, Older people, Rural health care

## Abstract

**Background:**

Admission to a nursing home is generally regarded as a stressful time for older people and their carers. Although the choice of home is significant in facilitating a more positive transition, few studies have explored this issue in detail, particularly in the context of rural communities. With a worldwide ageing population and an increasing demand for long-term care facilities, it is important to highlight the factors that can improve the experience of entry to long-term care and the role of nursing home staff in facilitating a more positive transition for older people and their families.

**Methods:**

The overall aim of this qualitative study was to explore rural family carers’ experience of the nursing home placement of an older relative. Semi structured interviews were conducted with 29 relatives of nursing home residents. Participants were selected from a large health and social care trust in the United Kingdom. Data were analysed using grounded theory principles and procedures and NVivo software.

**Results:**

Rural family carers had a strong sense of familiarity with the nursing homes in their area and this appeared to permeate all aspects of their experience. Carers who reported a high degree of familiarity appeared to experience a more positive transition than others. This familiarity was influenced by the high degree of social capital that was present in the rural community where the study was conducted. This familiarity, in turn, influenced the choice of nursing home and the responses of family carers. The theory that emerged suggests that familiarity was the key factor influencing rural family carers’ experience of the nursing home placement of an older relative.

**Conclusions:**

The population of the world is ageing and nursing homes are increasingly providing care to older people with multiple and complex needs. This study makes an important contribution to the ways in which the move to long term care can be managed more effectively by increasing awareness of the importance of familiarity, stability and social capital in the lives of older people and their carers.

## Background

The population of the world is ageing and this poses major challenges to health and social care systems [1]. Across Europe, current government policies focus on supporting older people to remain at home [2-4]. However, an increase in the number of people with chronic illness and dementia along with the costs associated with caring for these people at home, means that nursing homes will continue to play an important role in the provision of long-term care to older people [4].

There is a general consensus in the literature that admission to a nursing home is a stressful experience for older people and their relatives [5-7]. Research findings indicate that, for relatives, the experience is associated with conflicting emotions such as guilt, anger, sadness, relief and in the case of spouses a strong sense of loss and separation [7,8]. Despite the volume of literature on carers’ experiences of entry to care, few studies have investigated the factors influencing the choice of home. A review of the literature suggests that information about long-term care facilities is extremely limited and often reduced to a list of homes, further compounding the stress experienced by relatives [9,10]. Cheek and Ballantyne [11] found that prospective residents were often passive in the search and selection process and that this lack of planning left families devastated and overwhelmed by the decision they face on behalf of their older relative. A UK study used interviews to explore the experiences of 48 people who had assisted a close relative to move into a nursing home. The findings revealed three phases to the transition: making the best of it, making the move and making it better [8]. The authors concluded that participants were largely unprepared for the realities of nursing home life and that health and social care practitioners had enormous potential to influence relatives’ experiences of entry to nursing home care. These findings have been supported by other studies which explored the experience of entry to care from a temporal perspective [12,13].

A Canadian study used interviews (n = 29) to explore the ways in which family caregivers of institutionalised relatives with dementia perceived the relocation of their relative to a more home-like setting of care [14]. Findings pointed to the centrality of relationships in creating a truly home-like environment. The authors concluded that the creation of a meaningful home for persons with dementia must encompass a relational orientation, both philosophically and in practice that is inclusive of residents, staff and family. These findings were supported by research on the contribution of staff in developing relationships in long-term care settings which concluded that the development of a relationship–based approach to care routines had the potential to improve the experience of older people and their families [15]. Despite the growing body of literature on family caregiving and entry to care, there remains a limited volume of research that explores family carers’ experiences of nursing home placement in a rural setting and this study aimed to address this imbalance.

## Methods

The broad aim of this study was to explore rural family carers’ experience of the nursing home placement of an older relative with a focus on providing answers to the following questions:

•what factors influenced the choice of nursing home.

•who was involved in the decision making process.

•how did the move impact on family carers and, as they perceived it, their older relatives.

•what factors facilitated or hindered a successful transition from home care to nursing home care.

This qualitative study was conducted using grounded theory principles and procedures as described by Strauss and Corbin [16]. Ethical approval was granted by the University of Ulster Research Ethics Committee and the study adhered to the RATS guidelines on qualitative research. The study site was a large Health and Social Care Trust which covers some of Northern Ireland’s most isolated and deprived rural areas. Carers were selected for interview on the basis that they (1) were able and willing to participate in the study, (2) had fulfilled the role of primary carer, (3) had been involved in the move to the care home and (4) gave informed and written consent.

Prior to the commencement of data collection, the researcher met with the manager of the local Registration and Inspection Unit (an independent organisation responsible for regulating nursing homes) who provided a list of all the nursing homes in the study site (n = 20). This list comprised small and large homes most of which were privately owned by families (n = 13) with the remainder owned by large corporate organisations. Consistent with grounded theory methodology [16], purposive sampling was adopted in the initial phases of data collection. Thereafter theoretical sampling was employed. Purposive sampling is a sampling strategy in which participants are handpicked by the researcher, either because they are typical of the phenomenon of interest or because they are knowledgeable about the issues under investigation [17]. At the commencement of data collection, a sample of four homes was purposively selected to include a mixture of small/large, family/non-family owned homes located throughout the study site. Having made the selection, the manager of the Registration and Inspection Unit was asked to verify that the homes selected were an accurate reflection of the range, size and population of nursing homes within the area under investigation.

Recruitment strategies used by the Principal Investigator (AR) included newspaper advertisements, notices in nursing homes and direct contact with nursing home managers who administered participant information packs (containing an information sheet about the study and a reply slip) to residents’ relatives. The latter strategy was the most effective and resulted in the recruitment of 27 of the 29 participants. As data collection proceeded and the basis of a theory began to emerge, it became necessary to theoretically sample more non-family owned homes as tentative findings seemed to suggest something different about the experience of relatives in this type of home. Theoretical sampling involves selecting research participants on the basis of themes emerging from current data and analysis, in order to develop and elaborate categories and examine provisional hypothesis [18]. The remaining non-family owned homes were then invited to participate in the study. Similarly, if respondents mentioned anything that was considered to be of interest, this was followed-up in subsequent interviews. The process of theoretical sampling continued until the emerging concepts and categories reached saturation. The 29 participants were recruited from 10 of the 20 homes in the study site. Most of the carers had relatives in family owned homes (n = 23) and although the researcher tried to recruit cares from non-family owned homes to test the emerging theory, the response was disappointing.

Carers interested in the study were informed that their anonymity and confidentiality would be protected. Following verbal and written consent, participants were interviewed individually using a semi-structured format. Individual interviews were used because of the sensitivity of the topic while the semi-structured format provided the flexibility required in grounded theory methodology. The interview began with a broad question “*Can you tell me about the background to your relative*’*s nursing home placement*?. Although an interview guide was used in the initial interviews, this was modified as data collection and analysis progressed in order to pursue emergent themes consistent with grounded theory methodology. All interviews were audio recorded and transcribed with the consent of participants. Sampling continued until the emerging concepts and categories reached saturation.

### Data analysis

Data were subjected to three types of coding consistent with grounded theory principles [16]. Open coding was the process through which concepts were identified and their properties and dimensions rediscovered in the data. The initial transcripts were analysed on a line-by-line basis and a number of concepts emerged from this process. These concepts were then grouped into categories and sub-categories. The NVivo software package [19] greatly facilitated this process as it enabled concepts to be grouped to one or more categories until the theory began to emerge. Axial coding, termed axial because coding occurs around the axis of a category, was achieved by relating categories to sub-categories. Selective coding, the final stage of coding was the process of integrating and refining categories. They were then organised around a central explanatory concept or key category that appeared frequently in the data, related easily to other categories and possessed explanatory and theory generating power.

### Ensuring rigor

Various strategies were used to ensure the rigour of the study. The study was conducted with awareness and acceptance of the authenticity criteria described in the literature [20,21]. Equal access was promoted by providing accessible and comprehensive information to all participants about the nature of their involvement in the study. During the interviews and as appropriate, the researchers repeated what participants said to ensure confirmation of meaning and to ensure that the ‘truth’ of the participants’ experiences was accurately interpreted. An enhanced awareness of self and others was maximised during the interview process by introducing examples to clarify question and through the process of theoretical sampling which introduced divergent perspectives into the data collection process. The constant comparison of emerging data facilitated the verification of findings and minimised the likelihood of personal bias. Therefore, data emerging inductively were confirmed deductively by further theoretically sampled data. Concepts and categories that emerged repeatedly until saturation was reached were considered to have high levels of both truth and value. Concepts and categories that were not verified by subsequent data were considered to be lacking in truth and consistency and therefore were not included in the theoretical framework that emerged from the study. Theoretical sensitivity was maximised in a number of ways. Two research journals, one containing field notes and the other theoretical memos were used to record notes about the decision-making process throughout the study including sampling and analytical decisions. Thoughts and reflections about the interview process and its context including non-verbal behaviour and post interview discussions were also recorded.

To enhance the validity of the categorisation method and guard against researcher bias, two colleagues independently viewed a selection of original uncoded manuscripts and provided feedback on their interpretation. After the selective coding process, both colleagues were given copies of coding exemplars and the original transcripts from which these were drawn. Peer verification as a method of ensuring rigor has been criticised by researchers [22,23] who stated that it is unlikely that two people will interpret data in the same way because the production of themes and categories depends upon the unique creative processes between the researcher and the data. Others argued that the process of verification encourages thoroughness, adding that whether this is carried out by a conscientious researcher, by a team or by experts is immaterial, what matters is that a systematic process was followed [24].

## Results

A total of 29 carers participated in the interviews. Almost half were daughters (n = 14) and the others comprised sons (n = 6), wives (n = 3), daughter-in-laws (n = 2), nieces (n = 2) and nephews (n = 2). Dementia, stroke and mobility disorders were the main factors that precipitated the care home placement. The majority of carers (n = 27) resided close to their older relative’s nursing home.

The carers in this study were determined to keep their relative at home for as long as possible. However, there came a time when this was no longer an option because of further deterioration in the health of the older person and/or a reduction in the carers’ ability to cope. The move to a nursing home was influenced by the carers’ familiarity with the nursing home history, staff, residents and the local community. This familiarity was further influenced by a high degree of social capital and an efficient ‘*grapevine*’, (an informal person-to-person means of communication) which appeared to thrive in these small rural communities. As other aspects of the study (pertaining to research questions 2, 3 and 4) have been reported elsewhere [25,26], the findings presented in this paper will focus specifically on the core category that emerged from the study and the impact of familiarity on the choice of home and the success of the transition from the perspective of the family carers.

### The core category

In grounded theory, the central or core category represents the main theme of the research. At a simplistic level, it consists of the products of analysis reduced to a few words that explain what the research is all about. The core category or central phenomenon generated from this grounded theory study was the centrality of familiarity in rural family carers’ experience of the nursing home placement of an older relative. This ‘familiarity’ permeated all aspects of the placement. Its development as the core category will now be explained in detail with reference to narrative exemplars as appropriate.

### Familiarity with the nursing home history

Most of the older people in this study resided in family owned nursing homes and many carers had known the home-owners for several years. This sense of familiarity with the home by virtue of one’s acquaintance with the owners was captured in the following words of two daughters:

*I went to school with Mrs Trusdale and I knew her from then. I knew that she was a good person. I knew it would be fine.* (Interview 9)

Having access to the local grapevine, which appeared to thrive in the rural community where the study was based, further enhanced carers’ sense of familiarity with nursing homes in their area as evidence in the following quotations:

*It is very much orientated towards country people. That is the report I have heard about it* (Interview 14)

*I had heard a lot of reports about it….you know the way word gets around.* (Interview 5)

There were other examples, closely related to the familiarity issue where older people had some sort of historical connection to a particular home. In the following quotation, a son highlighted this connection:

*From she was a young woman she used to work in a well-known café during the war and there would have been a few priests she would have known in ‘The Cloisters’. When ‘The Cloisters’ nursing home was being built, she would often say, ‘If I ever need to go a nursing home, I’ll go there’......But I suppose it’s easy to say that at the time.* (Interview 18)

In contrast, familiarity was not a consideration for a minority of carers who resided outside the community:

Researcher: Looking back at it now, is there anything that would have made it easier for you?

*Interviewee: No, I am not sure at that stage. At the first stage it would have been useful to have more information. In the end, it was a matter of saying that it looks like any other nursing home and there is probably not a lot of difference apart from the level of care.* (Interview 4)

### Familiarity with nursing home staff

Because this study was conducted in a small community, many carers knew the staff in the nursing home long before their older relative was admitted and this was a source of great comfort to them and, as they perceived it, to their older relative. Some carers had family members or friends who worked in the nursing home and this greatly enhanced their sense of familiarity with the home.

*The lady who lived next door to her used to look after her when we went on holiday. She actually worked in the nursing home for a while and that is another reason why we got her to go to that home. She would have a familiar face there as well. She would call with her.* (Interview 15)

Continuity of care was an important issue for carers. In some cases, older people were being cared for by nursing assistants in the home who had also been involved in their care prior to the placement.

*Jayne (home help) had been here for 5 years helping me to put him to bed at night. Now, he looks forward to Jayne coming to chat with him.....it’s contact with home over there. She works in the home 4 nights one week and 3 nights the other. We knew the manager for years. She worked in the hospital whenever he was in for the stroke. We got to know all the ones in the home, most of the staff who were there when mummy was there five years ago.* (Interview 1)

*There are a couple of girls who would actually have been in the day centre where they knew Mummy which is a great help.* (Interview 19)

The geographical area where this study was conducted has a fairly stable population and all but two of the carers interviewed had lived there for many years. The following quotations emphasised the importance of familiarity with the nursing home staff, not only for carers, but also for the older person:

*I would have known quite a few of the nursing home staff. One of the girls started as a staff nurse. She would be a girl in her middle 40s. She is married these 20 years. When she walked in my mother said to her ‘I remember you when you were going to school’. She actually was right.* (Interview 21)

*She would go in and sit with the nursing staff because she chats with them about people in the town she knows.* (Interview 18)

The findings from this study revealed further examples of intergenerational contact, often characterised by role reversal whereby the care-giver had become the care recipient. This was depicted poignantly in the words of a wife:

*I know all the nurses and all the staff..... they’re all from around. There is a girl looking after Jack now and he looked after her as a child. She used to live beside us and she is a lovely girl. He used to look after her and now she is looking after him. That means a lot to me and to him. He knows who she is and she plays cards with him.* (Interview 11)

### Familiarity with nursing home residents

The sense of familiarity also extended to the carers’ knowledge of other residents and the comfort they took from feeling that their relative would have something in common with other residents because they were …’*all locals*’ (Interview 18). Carers in this study knew many of the residents in the nursing home and there was a strong sense of community spirit. This was evidenced by the fact some used to take their older relative to visit friends or neighbours in the home long before their own placement experience. It appeared that most of the homes in this study, perhaps because they were owned and managed by local people, remained an integral part of the community. People continued to visit friends and neighbours just as they would have done prior to the placement. The following quotations illustrated this point:

*I used to take her to see a friend who moved into the home and loved it and said it was the best thing she had ever done going to ‘The Glen’ and because mum is vaguely familiar with it, thinking about the fact that she can’t see perhaps that would be reassuring and it is very near. It only takes me 10 minutes to get there.* (Interview 2)

*As a rural community things are different. Obviously I would know people. In Belfast etc. nobody knows anybody whereas in the rural community you always know somebody.* (Interview 25)

In other cases, carers became familiar with the home and its residents as a result of respite arrangements.

*I had an aunt who was in the ‘Blueberry Hill’ for respite for about eight weeks and I always liked it. It was friendly and you could come and go as you pleased. I felt that ‘Blueberry Hill’ was a bit more homely as if you were moving from one home to another.* (Interview 16)

Knowing other residents and for those who were able to do so, communicating with other residents, was extremely important as the following words of a wife illustrated:

*He knows this farmer for years and the two of them talk about farming, the church and their families. Both are the one age. Lucky in one way to have someone there.* (Interview 1)

### Familiarity with the local community

Carers who resided in the local community, as the vast majority of respondents did, were also familiar with local health and social care practitioners and sought support from these individuals. This support took the form of validating the placement decision and acting as an advocate for the carer during the transition process. In most instances, the familiarity felt by carers was not limited to familiarity with the home, the staff or the residents but rather, a combination of some or all of these factors. This multiple familiarity was displayed in the following extract from an interview with a daughter-in-law:

Researcher: Why did you choose the ‘Cloisters?

*Interviewee: I would say it was because there was mass. She would have been a great one for praying and it is the nearest nursing home to her home as well. Maybe because I worked here, I don’t know. A bit of everything. She would know quite a few people here. I do think it was mainly because it was near home and there was mass.* (Interview 13)

Carers recognised the importance of having a sense of familiarity with the nursing home and this undoubtedly eased their transition. These multiple familiarities, whereby the carer and older person had links with the home, were evident in the following words of a nephew:

*I decided I wanted him to go to ‘The Limes’ because it is a good nursing home. I had a look about it. ‘The Limes’ is a nice home and it was comfortable and there were people in it he did know. He had cousins even working in it.* (Interview 28)

All of these findings suggest that while the nursing home placement was regrettable by virtue of its association with the declining health of the older person, carers used their familiarity with the home to ease the transition. Further, they believed that the familiarity that they had with the home extended to their relative who also experienced, in their opinion, a better transition than that which would have occurred in unfamiliar surroundings.

The two carers who lived away from home did not appear to experience the benefits of familiarity. However, it is possible that their overall experiences may have been influenced by their own personal circumstances. One carer was an only son and the other an only daughter whose two brothers had died some years ago. Both carers bore sole responsibility for their older relative and their overall experience of the transition was not positive. In the first extract, when asked if she had an input into the choice of home for her mother, the daughter replied:

*No……. I didn’t know where the nursing homes were or anything about them. I hadn’t a clue.* (Interview 10)

In the following quotation, a son reflected on the possibility that he may not have been as aware of the deterioration in his father’s condition by virtue of the fact that he was only able to visit at weekends:

*During that fortnight period that he had been in hospital I began to hear from some of the neighbours of all the things that had happened during the period before that. It became obvious that people out here had been looking after him and worrying about him for a while but didn’t want to say anything. I felt almost at that stage that I was getting the point of blame for not having responsibility for him. When we went down on the Sunday he didn’t appear to be too bad.* (Interview 4)

## Discussion

The findings of this study suggest that the experience of family caregiving and entry to care is influenced by cultural and geographical variables as epitomised by the centrality of familiarity in this study. Familiarity is defined as ‘*close acquaintance with or knowledge about something* ‘[27]. To date, familiarity has not been the subject of in-depth discussion in the literature on entry to care. Although some studies have acknowledged its importance in assisting carers to cope with the nursing home placement of an older relative [5-9], few have an elaborated on this issue in any great detail. However, the findings of this study suggest that at a time of crisis, familiarity takes on a new significance in terms of facilitating a more positive transition. Its significance therefore in the context of the literature on entry to care is notable by its virtual absence.

In this study, having a sense of familiarity with the nursing home staff, residents and history was a key factor influencing rural family carers’ experience of the nursing home placement of an older relative. Most of the respondents were drawn from family owned homes and the owners lived within the same community as residents, relatives and staff. Perhaps because of this, these homes appeared to be perceived as extensions of the community, a sub-culture within a culture, described in the literature as small and active communities within a wider community context [28]. This is supported in a Canadian study which highlighted the centrality of relationships in creating a homelike environment for people with dementia [14].

‘*Working in the dark*’, characterised by relatives not knowing where to start in the search for a nursing home, emerged as a key finding elsewhere [5] and is a recurrent theme in the literature on entry to care. However, it was not a major issue for carers in this study, most of whom were very much ‘*working in the know*’. Because carers and their older relatives had been part of the same community, they appeared to have had knowledge of and familiarity with local nursing homes and the community in general. In this way, the study site shared many of the rural characteristics described by Williams and Cutchin [29] in that it was a close knit community characterised in the main by close rather than impersonal relationships, good communication structures and a sound community spirit.

The findings of this study resonate with the growing body of international literature on social capital. The concept of social capital has captured the interest of many researchers in different fields such as public health, medical sociology and education over the past two decades [30]. Social capital is defined as “the stock of active connections among people: the trust, mutual understanding and shared values and behaviours that bind the members of human networks and communities and make cooperative actions possible” [31], p.4. Social capital therefore benefits people by instilling a sense of belonging and establishing relationships of trust and tolerance [32]. There is evidence to suggest that close personal relationships and network ties have a positive impact on health and well-being and that people whose lives are rich in social capital cope better with trauma [33,34]. The issue of social capital has also been addressed by researchers exploring ways of developing community in care homes and it has been suggested that care homes as communities is a model where the needs of all stakeholders can be reconciled and met as long as all parties recognise the contribution each has to make [25,35,36].

Accepting that social networks, trust and a sense of community are key factors in the development of social capital, it appears that most of the carers in this study were rich in this resource which in turn facilitated the exchange of information about nursing homes and provided emotional support during the care home placement, an event which is generally considered to be an extremely difficult. Putnam [32] also argued that the networks that constitute social capital also serve as conduits for the flow of helpful information. This offers a more elaborate explanation about the importance of familiarity in enabling the carers in this study to elicit the necessary information about nursing homes in their area. It also explains why the two carers who lived outside the community and consequently were not in a position to benefit from social capital, found themselves ‘working in the dark’ when faced with the task of choosing a nursing home for their relative. This contrasted sharply with the majority of rural carers who were clearly ‘working in the know’ by virtue of the social capital derived from their community network.

It is widely acknowledged that rurality is more than a geographical concept but instead should be viewed as a combination of social practices and structures [37]. A literature search revealed little by way of the significance of rurality on carers’ experiences of the nursing home placement of an older relative. An American study distinguished between two circles of care that exist in rural communities: *formal circles of care*, comprising health and social care professionals and *informal circles of care* comprising family, friends and neighbours [38]. Others recognised the unique features of rural living and the importance of relationships [39-41]. Glover [39] argued that because rural care providers have more knowledge and consequently more at stake than urban care providers, a lack of support not only lets the profession down but perhaps more importantly lets the community down as well. This in turn exerts an impact on practice as patients and health professionals have to live with the consequences of decisions. This view is supported by Lauder *et al*. [41], who noted that rural nurses deal with a lack of anonymity and much greater role diffusion than nurses in urbanised areas. The disadvantages of rurality including transportation, isolation and access to services have been widely reported in the literature [42-44]. However, the findings of this study suggest that rurality had clear benefits for care home residents and their relatives. Because the number of care homes was limited, the ‘*local*’ home was the one where most older people went, where friends and relatives worked and where neighbours and relatives visited, thereby further easing a difficult transition.

The theory that emerged from this grounded theory study suggests that rural family carers who have a sense of familiarity with a nursing home’s history, residents, staff and the local community have a more successful transition and less negative overall experience of an older relative’s entry to care than carers who lack this sense of familiarity. Although there have been many studies on entry to care, there is a dearth of research on rural carers' experience of the nursing home placement of an older relative. Because little is known about this particular aspect of entry to care, the theory generated offers new insights into rurality and entry to long-term care.

**Figure 1:**
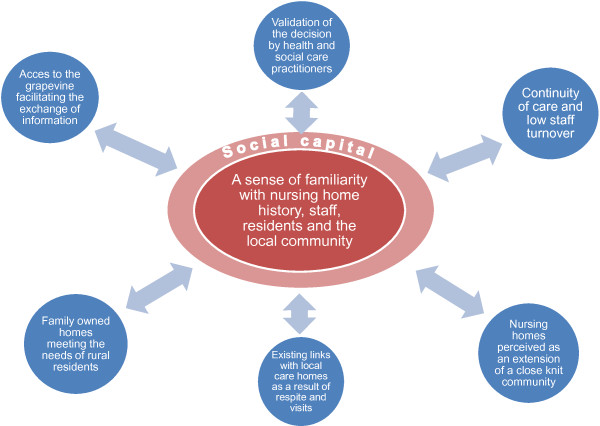
**Conceptual model of the factors influencing rural family carers’ experience of the nursing home placement of an older relative.**

The core category and its component parts are described in a conceptual model of the factors influencing rural family carers’ experience of the care home placement of an older relative (Figure 1). Carers’ sense of familiarity with the nursing home history, staff, residents and the local community was clearly influenced by the high degree of social capital reported in this study. This social capital in turn facilitated carers to develop positive relationship with health and social care practitioners who subsequently acted as advocates in validating the placement decision. Continuity of care and the low staff turnover in local nursing homes further enhanced social capital as people formed relationships and got to know one another. Living in a close knit community, carers perceived their local nursing home as an extension of the community. This eased the transition which was also influenced by carers’ existing links with the home as a result of respite arrangement or having previously visited the home. Familiarity and social capital were further influenced by family owned homes which met the needs of rural residents and by access to the ‘*grapevine*’, which facilitated the exchange of information. The two-way arrows used in Figure 1 illustrate the link between social capital and its influencing factors. Social capital was enhanced as a result of existing links with local care homes and equally these links and networks further enhanced social capital. Similarly, social capital facilitated easy access to the local knowledge which in turn generated more social capital. However, carers who resided outside the rural community and consequently lacked social capital, did not experience this sense of familiarity and reported less positive experiences of their older relatives’ placement.

The findings of this study emphasise the importance of familiarity, continuity, stability and social capital in the lives of older people. While familiarity, for better or worse, is an integral part of life in rural communities, consideration should be given to cultivating more familiar surroundings in less rural settings. This has implications for care home staff who could take a more proactive role in achieving or enhancing familiarity by supporting residents to forge relationships with other residents and their families. This necessitates the collection of biographical information from residents so that this can be used to make connections with other residents. Even very tenuous connections pertaining to residents’ education or employment history can be used effectively to construct familiarity and in doing so to facilitate a wider social intercourse than would otherwise be possible.

### Limitations of the study

As most of the carers were recruited through nursing home managers, it is not possible to identify the number of people who refused to participate in the study and this is acknowledged as a potential limitation. It is possible that managers may have deliberately selected carers who had positive experiences of entry to care. It is also possible that the carers who volunteered were biased in some way although the range of responses suggests that they spoke openly and frankly about their overall experience. While the carers in the study appeared to be rich in social capital, this was not explicitly addressed in the interviews. Most of the carers in this study had relatives in family owned nursing homes and it is possible that this may have had a bearing on the findings. This was explored further by theoretically sampling non-family owned homes but the response was disappointing. One can only speculate on the reason for such a disappointing response but a credible explanation may be related to the autonomy of family owned home managers who were also the home owners. This decision-making autonomy may not have been shared by the managers in other homes who may have had concerns about the consequences of negative comments on their managerial positions.

## Conclusion

Recognising that nursing homes are increasingly providing care to older people with multiple and complex needs, this study makes an important contribution to the ways in which the move to long term care can be managed more effectively by increasing awareness of the importance of familiarity, stability and social capital in the lives of older people and their carers. In most countries in the world the proportion of older people in local populations is higher in rural areas than in urban areas [45]. Despite this, the literature on entry to care appears to reflect an urban bias with the experience described in predominantly negative terms. A rural model for health and social care provision is recommended and the theory described in this study contributes to our understanding of the different way in which entry to care is experienced in rural populations. Rather than clustering nursing homes in certain areas, a wider distribution of small homes in rural communities would facilitate people to maintain their community connections and to ‘age in place’. Further research is required to test the theory described.

## Competing interests

The authors declare that they have no competing interests.

## Authors’ contributions

AR had the original idea, conducted the interviews with participants and prepared the first draft. Both authors developed the proposal, conducted the analysis, read and approved the final manuscript. Both authors read and approved the final manuscript.

## Authors’ information

Both authors have professional qualifications in adult and mental health nursing. AR leads the ‘*Care Provision for Older People*’ strand of the Person-Centred Practice Research Centre at the University of Ulster and HMcK is Pro-Vice-Chancellor (Research and Innovation). AR’s specific research interests include the needs and experiences of family carers, implications of nursing and residential care for older people and their families and community care for older people in rural locations. Both authors are full members of the Institute of Nursing Research which was ranked 3rd in the UK in the last Research Assessment Exercise with 100% of its research activity considered to be of an international standard and 40% world leading.

## Pre-publication history

The pre-publication history for this paper can be accessed here:

http://www.biomedcentral.com/1472-6963/13/252/prepub
